# GluR2 overexpression in ACC glutamatergic neurons alleviates cancer-induced bone pain in rats

**DOI:** 10.1186/s10020-025-01183-9

**Published:** 2025-04-07

**Authors:** Futing Ba, Jinrong Wei, Qi-Yan Feng, Chen-Yang Yu, Meng-Xue Song, Shufen Hu, Guang-Yin Xu, Hai-Long Zhang, Guo-Qin Jiang

**Affiliations:** 1 Department of General Surgery, The Second Affiliated Hospital of Soochow University, 1055 San-Xiang Road, Suzhou, 215004 China; 2 Laboratory for Translational Pain Medicine, Institute of Neuroscience, Soochow University, Suzhou, 215123 China; 3 Center of Translational Medicine and Clinical Laboratory, The Fourth Affiliated Hospital of Soochow University, Suzhou, 215123 China

**Keywords:** Cancer-induced bone pain, Anterior cingulate cortex, Glutamatergic neurons, AMPA receptors, GluR2

## Abstract

**Background:**

Cancer-induced bone pain (CIBP) is a complex chronic pain with poorly understood mechanisms. The anterior cingulate cortex (ACC) plays a critical role in processing and modulating chronic pain. This study investigates how the GluR2 receptors (calcium impermeable AMPA receptors) in ACC glutamatergic neurons regulate CIBP.

**Methods:**

The CIBP models were established by injecting Walker 256 cells into the tibia of SD rats. Paw withdrawal threshold (PWT) and paw withdrawal latency (PWL) were used as indicators of hyperalgesia. The immunofluorescence staining was employed to detect the expression of c-Fos in ACC and identify the subtypes of co-labeled c-Fos^+^ neurons. Real-time monitoring of calcium activity in ACC glutamatergic neurons was achieved through the fiber photometry. The excitability of glutamatergic neurons in ACC was modulated using chemicalgenetics and optogenetics techniques. The expression of GluR2 at the mRNA and protein level in ACC were assessed using RT-qPCR and Western blotting.

**Results:**

There were significant reductions in PWT and PWL of CIBP rats after Walker 256 cell injection. The ACC of CIBP rats showed increased c-Fos expression compared to sham rats, with mainly activated c-Fos co-localized with glutamatergic neurons. Optogenetic or chemogenetic activation of ACC glutamatergic neurons led to increased hyperalgesia in sham rats, while suppression of their activity alleviated hyperalgesia in CIBP rats. Calcium activity in ACC glutamatergic neurons of CIBP rats was increased with suprathreshold stimulation of von Frey filament. Notably, surface GluR2 protein and mRNA were reduced in ACC of CIBP rats. Furthermore, overexpression of GluR2 by AAV-CaMKII-GluR2 injection was decreased c-Fos expression in ACC and alleviated hyperalgesia in CIBP rats.

**Conclusions:**

These findings suggest that decreased surface GluR2 receptors in ACC glutamatergic neurons contribute to calcium activity and excessive excitability, thereby inducing CIBP in rats. Conversely, GluR2 overexpression in ACC glutamatergic neurons alleviates CIBP in rats. This study provides a new potential therapeutic approach for targeting the GluR2 receptor to alleviate CIBP for cancer patients.

## Introduction

Cancer-induced bone pain (CIBP) is a persistent and worsening pain resulting from the cancer metastasis to the spinal cord cavity or bone tissues (Yang 2023). In breast cancer cases, about 75% involve bone metastasis, with a 5-year survival rate of 22.8% (Macedo et al. 2017). Skeletal-related events (SREs), including CIBP, affect up to 43% of patients with bone metastases, with 75% of advanced breast cancer patients experiencing SREs (Gillespie et al. 2023). Patients with CIBP often face a persistent pain that worsens gradually, with unpredictable episodes of breakthrough pain, especially during activities like walking, exercise, and at night (Muralidharan and Smith 2013), This pain significantly impacts their quality of life and can worsen symptoms of depression and anxiety (Michaelides and Zis 2019). Despite systemic treatment, around 50% of CIBP patients still struggle with inadequate pain control and poor response to therapy (Sulistio et al. 2023; Michael et al. 2023). The traditional'step analgesia'approach, while effective in managing pain, does not prevent fractures, maintain mobility, or extend survival, and can have side effects (Zajączkowska, et al. 2019; Desandre and Quest 2009). Therefore, there is a critical need for new therapies to provide palliative care for cancer patients, easing pain and improving their quality of life.

The Anterior cingulate cortex (ACC), which encompasses Brodmann areas 24, 32, and 33, is a critical component of the limbic system in the brain (Gasquoine 2013). It functions as a central hub for pain regulation (Ntamati et al. 2023) and is involved in emotional processes like fear (Bian et al. 2019), empathy (Li, et al. 2021; Smith et al. 2021a), cognition (Brockett et al. 2020), and reward behavior (Young, et al. 2023). Resting-state functional magnetic resonance imaging (rs-fMRI) has revealed abnormal activation in the cingulate, prefrontal cortex, and ventral striatum as part of the central nervous system's regulation of CIBP (Buehlmann et al. 2018). Recent studies have revealed that the ACC-NAc circuit (the neural circuit in which anterior cingulate cortex neurons project to nucleus accumbens neurons) is specifically linked to empathic responses related to pain and analgesia, emphasizing the connection between the ACC and human pain empathy (Smith et al. 2021b). According to a study, increased neuronal activity in the ACC contributes to the maintenance of bone cancer-induced mechanical hypersensitivity and suggests that the ACC may serve as a potential therapeutic target for the treatment of bone cancer pain (Chiou et al. 2016). Nevertheless, the precise mechanism by which pain stimulation triggers neuronal activity in the ACC and perpetuates the development and progression of CIBP remains unclear.

Ionotropic glutamate receptors (iGluRs) are widely distributed in the brain, spinal cord, and peripheral nervous system. They act as ligand-gated ion channels, responding to glutamate release from presynaptic terminals. These receptors are crucial for excitatory synaptic transmission and synaptic plasticity in the central nervous system, contributing to various neurological disorders (Hansen et al. 2021). iGluRs are categorized into N-methyl-D-aspartate (NMDA), α-amino- 3-hydroxy- 5-methyl- 4-isoxazole-propionic acid (AMPA), and kainate (KA) receptors (Fu et al. 2019). The AMPA receptors consist of subunits GluR1, GluR3, and GluR4 with Ca^2+^ permeability and GluR2 lacking Ca^2+^ permeability (Zhou et al. 2022). Following peripheral nerve injury, there are lasting changes in excitatory synaptic transmission in ACC glutamatergic neurons, leading to increased presynaptic glutamate release and enhanced postsynaptic glutamatergic AMPA receptor response, these changes are implicated in the maintenance of pain (Xu et al. 2008). In a model of peripheral inflammatory pain hypersensitivity, it was observed that GluR2-containing AMPA receptors transitioned to a GluR2-lacking form, resulting in heightened Ca^2+^ permeability mediated by AMPA receptors at synapses in spinal dorsal horn neurons, this process contributes to the development and persistence of peripheral inflammatory pain hypersensitivity (Park et al. 2009). These findings suggest that alterations in AMPA receptor subunits on postsynaptic neurons may be involved in maintaining pain sensitization. However, the specific mechanism by which GluR2-containing AMPA receptors regulate and sustain CIBP through ACC neurons remains unclear.

Based on previous studies, we hypothesized that CIBP would activate ACC glutamatergic neurons, causing downregulation of AMPA receptors containing GluR2 on the postsynaptic membrane. This downregulation would result in increased permeability of the postsynaptic membrane to Ca^2+^, excessive excitability and hyperalgesia in rats with CIBP. The study may contribute to a better understanding of the mechanisms underlying hyperalgesia in CIBP, and potentially identify a clinical therapeutic target for managing cancer patients for pain relief.

## Materials and methods

### Animals

Female Sprague–Dawley rats weighing 180–200 *g* were housed in groups of four per cage in temperature- (24 ± 1 °C) and light-controlled (12-h light/dark cycle) room with access to standard rodent chow and water ad libitum. Animals were randomly assigned to different groups, and experiments were performed in blind manner. All procedures were approved by the Institutional Animal Care and Use Committee of Soochow University (SYXK 2022–0043). Animal experiments were conducted in strict accordance with the International Association for the Study of Pain (IASP).

### Rat model of cancer-induced bone pain

Walker 256 cells are a well-known rat breast cancer cell line that has been widely used in cancer research, implantation of Walker 256 cells was performed as previously described (Sun, et al. 2020; Kong et al. 2017; Wei et al. 2017; Zhou et al. 2015). Briefly, Walker 256 cells were grown in SD rats (60–80 g) by intraperitoneal injection of 2 × 10^7^ cells. One week later, the ascites was collected and resuspended with normal saline (1 × 10^8^ cells/mL). After establishing isoflurane anesthesia, a small incision was made in the right leg of SD rats exposing the tibial plateau. A hole was drilled into the tibia cavity, and 10 μL tumor cells or normal saline (NS) was slowly injected into the hole using a 10 μL microinjection syringe. To prevent leakage of cells outside the bone, the injection site was closed with bone wax. The incision was then sutured, and the rats were transferred to a warm pad to wake.

### Pain behavioral assessments

The measurement of paw withdrawal threshold (PWT) and paw withdrawal latency (PWL) described by our previous report (Sun, et al. 2020; Kong et al. 2017; Wei et al. 2017). In brief, the rats were habituated to a transparent Plexiglas box for 30 min. PWT was assessed using the von Frey up-down method. A series of calibrated von Frey filaments (VFF, Stoelting, USA, ranging from 1.0 to 26.0 g) were applied perpendicularly to the plantar surface of the hind-paw with sufficient force to bend filaments for 3 to 5 s. Each trial was repeated 5 to 6 times at 5-min intervals, and the mean value was used as the force to produce withdrawal response. The VFF force was recorded and the corresponding log threshold (g) values (Table 1) were used for subsequent data analysis. PWL was measured by heat radiation method for the thermal hyperalgesia of rats. A radiant heat source was focused onto the plantar surface of the hind-paw. Measurements of PWL were taken by an automatic timer that was started by activation of a heat source and stopped when withdrawal of the paw was detected with a photodetector. A maximal cutoff time of 20 s was used to prevent unnecessary tissue damage. The interval between the two tests was more than 5 min, and the test was repeated 5 times for each rat.

**Table 1:** The VFF force was recorded and the corresponding log threshold (g) values

von Frey filements force (g)	log threshold (g)
1.0	1.61
1.4	1.98
2.0	2.74
4.0	4.87
6.0	7.37
8.0	11.42
10	15.76
15	20.00
26	26.00

### Immunofluorescence staining

Rats were transcardially perfused with 0.9% normal saline and 4% paraformaldehyde (PFA) under deep anesthesia at 14 days after induction of the CIBP model. The brains were removed and immersed for post-fixation in PFA at 4 ℃ for 12 h. After dehydration with sucrose gradient, brains were embedded in OCT and then cut into sections with 25–30 μm thickness. Brain sections were washed with PBS and blocked with blocking buffer containing 7% normal donkey serum, 0.3% Triton X- 100, and 0.05% sodium azide for 1 h at room temperature. After blocking, brain sections were incubated with primary antibodies solution diluted with donkey serum blocking solution overnight at 4 ℃. The following primary antibodies were purchased from commercial suppliers: anti-Mouse-c-Fos (Santa Cruz, 1: 200, sc- 271243), anti-Rabbit-Glutamate (Sigma Aldrich, 1: 200, G6642), anti-Mouse-CaMKII (Cell Signaling Technology, 1: 100, 6G9), anti-Rabbit-GABA (Santa Cruz, 1: 200, A2052), anti-Mouse-NeuN (Merck Millipore, 1: 50, MAB377B), anti-Rabbit-GluR2 (Abmart, 1: 200, T57063), anti-Mouse-GFAP (Cell Signaling Technology, 1: 100, #3670), anti-Goat-Ib1 (Abcam, 1: 200, ab289874). After washing with PBS 3 times (10 min each time), the brain sections were incubated with secondary antibodies for 1 h at room temperature. The following secondary antibodies were purchased from commercial suppliers: Alexa Fluor TM 488 Donkey Anti-Rabbit IgG (Thermo Fisher Scientific, 1: 500, A21206), Alexa Fluor TM 555 Donkey Anti-Mouse IgG (Thermo Fisher Scientific, 1: 200, A31570), Alexa Fluor TM 555 Donkey Anti-Goat IgG (Thermo Fisher Scientific, 1: 200, A21432). We selected the same regions of the ACC in different rats for evaluation based on the location of the brain mapping, and captured images derived from the ACC region framed by the dotted line and analyzed them to reduce individual differences. We quantify it manually using a double-blind method (n = 4).

### Stereotaxic virus injection and optical fiber implantation

Following deep anesthesia, rats were head fixed on a stereotaxic apparatus (RWD Life Science, China, 71,000-M) with a bite bar and ear bars. After disinfection with iodophor solution, rat scalps were cut with a longitudinal midline incision to expose the skull. The skull above the target left ACC was removed carefully with a skull drill. The virus injection was made via a micro syringe (Gaoge, China) modified with glass micropipettes pulling by a Sutter Instrument P- 97 micropipette puller. 300 nL of the virus was injected into the left ACC of the rat brain using a microsyringe pump (Longer Pump, China, TJ- 2 A) at a rate of 30 nL/min. Front fontanelle as the origin of coordinates, virus injection coordinates are AP, + 1.0 mm; ML, + 0.5 mm; DV, − 2.5 mm. To prevent virus backflow, the micropipette was left in place for approximately 10 min after virus injection and then retracted from the brain slowly. Optical fiber implantation was conducted immediately after virus injection. The optical fiber (diameter, 400 μm, Newdoon, China) were secured to the skull of each rat using 3–4 screws and dental cement. The viruses used in this study are as follows: AAV2/9-CaMKII-hM3D(Gq)-mCherry (from BrainVTA Wuhan, China, titer: 5.40 × 10^12^ genome copies/ml); AAV2/9-CaMKII-hM4D(Gi)-mCherry (from BrainVTA Wuhan, China, titer: 2.44 × 10^12^ genome copies/ml); AAV2/9-CaMKII-ChR2-mCherry (from Gene Biotechnology, Shanghai, China, titer: 5.20 × 10^12^ genome copies/ml); AAV2/9-CaMKII-eNpHR-eGFP (from Gene Biotechnology, Shanghai, China, titer: 6.27 × 10^12^ genome copies/ml); AAV2/9-CaMKII-eGFP (from Gene Biotechnology, Shanghai, China, titer: 5.33 × 10^12^ genome copies/ml); AAV2/9-CaMKII-mCherry (from Gene Biotechnology, Shanghai, China, titer: 5.33 × 10^12^ genome copies/ml); AAV2/9-CaMKII-GCaMP6f (from Taitool Bioscience, Shanghai, China, titer: 3.14 × 10^12^ genome copies/ml); AAV-GluR2 and AAV-NC (GenePharma, China, titer: 1.37 × 10^12^ genome copies/ml).

### Optical stimulation

One week prior to modeling, the CIBP group had been injected with AAV2/9-CaMKII-eNPHR-eGFP or AAV2/9-CaMKII-eGFP into the left ACC, with CON group injected with AAV2/9-CaMKII-ChR2-mCherry or AAV2/9-CaMKII-mCherry. The behavioral tests were performed at 14 days after induction of the CIBP model. The delivery of a 20-s pulse of blue (473 nm, 2–5 mW, 20-ms pulses, 10 Hz) or yellow (594 nm, 3–5 mW, constant) light was controlled by optogenetic system (Alpha Omega Engineering, Israel).

### Chemogenetic Manipulations

One week prior to modeling, the CIBP group had been injected with AAV-CaMKII-hM4D(Gi)-mCherry (from BrainVTA, China, titer: 2.44 × 10^12^ genome copies/ml) into the left ACC, with CON group injected with AAV-CaMKII-hM3D(Gq)-mCherry (from BrainVTA, China, titer: 5.40 × 10^12^ genome copies/ml). At 14 days after induction of the CIBP model, the behavioral tests were measured before and at various time points (0.5, 1, 2, 4, 6, and 8 h) after interperitoneal injection of clozapine-N-oxide (CNO, 3 mg/kg, BrainVTA, China).

### Fiber photometry system

AAV-CaMKII-GCaMP6f (from Taitool Bioscience, China, titer: 3.6 × 10^12^ genome copies/ml) was injected into the left ACC of the SD rats. GCaMP consists of an enhanced green fluorescent protein (eGFP) fused to calmodulin (CaM) and myosin light-chain kinase (M13) (Luo et al. 2020). CaM is a target of Ca2^+^ within the cell can bind to Ca^2+^ (Chin and Means 2000).This binding is believed to cause a conformational change in eGFP, transforming eGFP into a more efficient configuration. Fiber photometry system (ThinkerTech Nanjing Bioscience Inc.) allows for real-time excitation and recording of fluorescence from GCaMP in freely moving rats. Two excitation wavelengths, 405 and 470 nm, were used in this system, coupled into a 400 µm optical fiber by a commutator. The laser intensity was adjusted at the tip of the optical fiber to 10 to 20 mW. Recording of calcium responses to VFF stimulation in awake rat employed fluorescence values obtained 2 s before (− 2 to 0 s) and 10 s after (0–10 s) stimulation. Data were classified based on behavioral events in individual experiments, and each stimulus was repeated eight times to ensure the accuracy and authenticity of the data. The photometry data were analyzed with custom-written MATLAB codes (MATLAB R2017b, MathWorks) using ΔF/F 2 s before stimulation as the baseline. The value of ΔF/F characterizes the change in fluorescence intensity around the event.

### Real-time quantitative polymerase chain reaction for mRNA analysis

Total RNA was extracted from the left ACC from the CON and CIBP rats using TRIzol (Ambion), and cDNA was synthesized from total RNA using an EasyScript One-Step gDNA Removal and cDNA Synthesis SuperMix kit (TransGen Biotech, China) following the manufacturer’s instructions. The expression level of mRNAs was normalized by the Ct value of GAPDH using the 2^∆∆Ct^ relative quantification method. The mRNAs of GluR1, GluR2, GluR3, GluR4 and GAPDH (internal control) were measured in the quantitative polymerase chain reaction using the following primers:

GluR1 forward primer: 5′-AATGTGGCAGGCGTGTTCTA- 3′,

reverse primer: 5′-GGATTGCATGGACTTGGGGA- 3′;

GluR2 forward primer: 5′-GCCAGAGTCCGGAAATCCAA- 3′,

reverse primer: 5′-CCGCACTCTCCTTTGTCGTA- 3′;

GluR3 forward primer: 5′-AGCCGTGCGATACGATGAAA- 3′,

reverse primer: 5′-ATAGAACACGCCTGCCACAT- 3′;

GluR4 forward primer: 5′-TACGACAAAGGAGAATGTGGCAG- 3′,

reverse primer: 5′-CAATGACAGCCAATCCCGAA- 3′;

GAPDH forward primer: 5′-TGGAGTCTACTGGCGTCTT- 3′,

reverse primer: 5′-TGTCATATTTCTCGTGGTTCA- 3′.

### Extraction of membrane protein and Western blotting

Expressions of GluR1, GluR2, GluR3, GluR4, GAPDH and Na^+^-K^+^-ATPase in left ACC from CON and CIBP rats were measured using western blotting. According to the manufacturer’s instructions, membrane proteins were extracted by the Mem-PER™ Plus Kit (Thermo Fisher Scientific, USA, 89,842). Supernatants of ultrasonic disruption were carefully prepared and the protein concentration was measured using a BCA Protein Quantitation Kit (Beyotime, China, P0010S). A total of 20–40 μg of the supernatant was taken in a centrifuge tube and made upto a uniform volume with normal saline. Add 5 × loading buffer (Beyotime, China), centrifuge briefly and denature at 75 °C for 10 min in a thermostatic water bath. The electrophoresis gel should be configured using the One-Step PAGE Gel Fast Preparation Kit-Box2 kit (Vazyme, China). The protein solution was loaded into polyacrylamide gels and fractionated on polypropylene electro phoresis (Bio- Rad), then transferred to polyvinylidene difluoride (PVDF, Merck Millipore) membranes at 200 mA for 2 h. The PVDF membranes that had been transferred were placed in a Tris- HCL buffer solution containing 5% fat- free milk at room temperature for 2 h and in cubated with primary antibodies for 24 h at 4 °C. The primary antibodies in the present study included anti-Rabbit-GAPDH (Goodhere, China, 1: 1000, AB-P-R001), anti-Rabbit-GluR1 (Abmart, China, 1: 2000, T55801), anti-Rabbit-GluR2 (Abmart, China, 1: 2000, T57063), anti-Rabbit-GluR3 (Abmart, China, 1: 2000, PK89136), anti-Rabbit-GluR4 (Abmart, China, 1: 2000, T58347), Anti-Sodium Potassium ATPase (Abcam, USA, 1: 2000, ab76020). After wash, the PVDF membranes were incubated with secondary antibodies for 2 h at room temperature. The secondary antibody in the present study included anti-rabbit peroxidase-conjugated secondary antibody (Jackson ImmunoResearch Laboratories, USA, 1: 2000). After wish, a drop of developer (NCM biotech, China) was added to the PVDF membrane, after which the ultra-high sensitivity gel imager (Bio-Rad, USA) was activated for imaging. The densities of protein bands were analyzed using Image J (National Institutes of Health, Betheseda, MD).

### Data analysis

All data were analyzed using GraphPad Prism 9.0 and MATLAB software. All data are presented as mean values ± SEM. Normality was checked for all data before comparison. The *t*-test was used to determine significance of changes between 2 groups. Two-way repeated-measures analysis of variance (ANOVA) followed by Sidak’s multiple comparison test were performed where appropriate. *p* < 0.05 was regarded as statistically significant.

## Results

### Injection of Walker 256 cells into the tibia cavity induced mechanical and thermal hyperalgesia accompanied by an increased in c-Fos expression in ACC

**Fig. 1:**
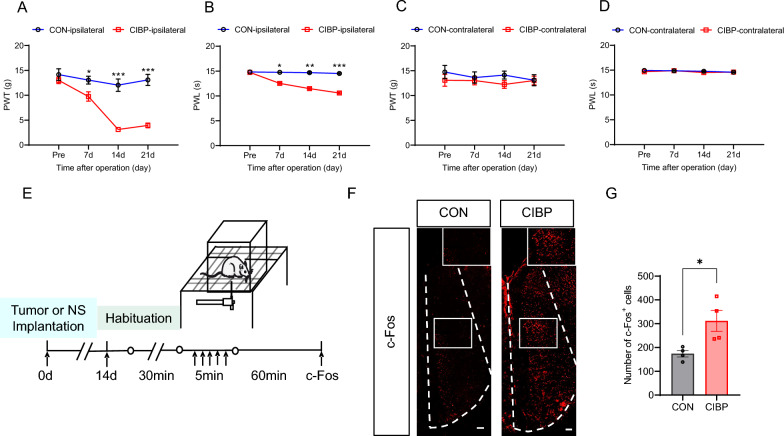
CIBP rats exhibited pain hypersensitivity with upregulation of c-Fos expression in ACC. **A**, **B** Compared with CON group, PWT and PWL in the ipsilateral hind-paw of the CIBP group were decreased at 7 d, 14 d and 21 d after modeling (CON, n = 8; CIBP, n = 11, *p < 0.05, **p < 0.01, ***p < 0.001, two-way ANOVA followed by Sidak’s multiple comparison test). **C**, **D** Compared with CON group, PWT and PWL in the contralateral hind-paw of the CIBP group were not different at 7 d, 14 d and 21 d after modeling (CON, n = 8; CIBP, n = 11, *p* > 0.05, two-way ANOVA followed by Sidak’s multiple comparison test). **E** Schematic representation of c-Fos expression evoked by von Frey filament stimulation. **F** Representative images of c-Fos^+^ cells in left ACC after von Frey filament stimulation, Scale bar: 100 μm, 10 × magnification. **G** Number of c-Fos^+^ cells in left ACC after von Frey filament stimulation (n = 4 brain sections from 4 rats for each group, *p < 0.05, two-tailed t-test)

The paw withdrawal threshold (PWT) and paw withdrawal latency (PWL) were measured before modeling (Pre), 7 d, 14 d and 21 d after modeling to examine whether Walker 256 cell injection induces pain hyperalgesia. The control group (CON) was injected with normal saline. The results showed that the PWT and PWL in the ipsilateral hind-paw of the CIBP group were lower than that in the CON group at 7 d, 14 d and 21 d after modeling (Fig. 1A, B). However, there was no significant difference in the contralateral PWT and PWL between the CON and CIBP groups (Fig. 1C, D). These data showed that injection of Walker 256 cells into the tibia cavity induced mechanical and thermal hyperalgesia in rats, consistent with previous studies (Sun, et al. 2020; Kong et al. 2017; Wei et al. 2017). To investigate whether the ACC is involved in CIBP, we compared the expression of c-Fos in the ACC after rats were given suprathreshold stimulation using von Frey filament. The steps of suprathreshold stimulation are shown in Fig. 1E. The results showed that the number of c-Fos^+^ cells was dramatically increased in the ACC of CIBP group as compared with CON group (Fig. 1F, G). These results suggested that increasing neuronal activity in the ACC may be involved in the development and maintenance of CIBP in rats.

### ACC glutamatergic neurons were the main type of neurons involved in CIBP

**Fig. 2:**
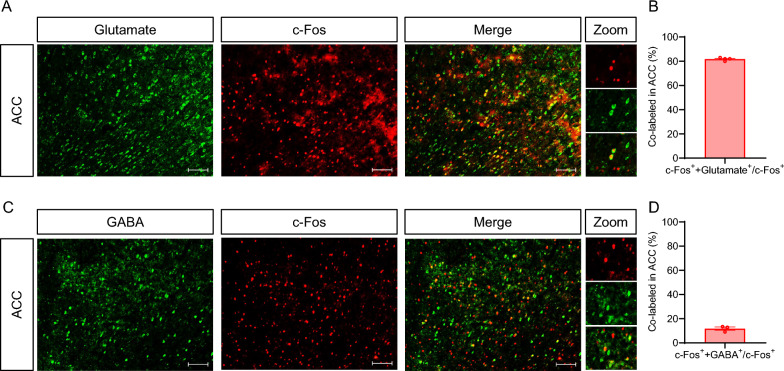
CIBP mainly activated ACC glutamatergic neurons. **A** Representative images of c-Fos (red) and Glutamate (green) co-expression in ACC of CIBP rats, Scale bar: 100 μm, 10 × magnification. **B** Percentage of co-expression of glutamate and c-Fos in ACC (n = 4 brain sections from 4 rats). **C** Representative images of c-Fos (red) and GABA (green) co-expression in ACC of CIBP rats, Scale bar: 100 μm, 10 × magnification. **D** Percentage of co-expression of GABA and c-Fos in ACC (n = 3 brain sections from 3 rats). The c-Fos was expressed in the nucleus, whereas Glutamate and GABA were expressed in the cytoplasm

To explore which type of neurons in the ACC were involved in CIBP, we double-labeled c-Fos^+^ cells with the glutamatergic neuron marker Glutamate and GABAergic neuron marker GABA. Immunostaining of c-Fos two week after CIBP showed that c-Fos^+^ cells activated by CIBP were mainly co-localized with glutamate (Fig. 2A), whereas only a few c-Fos^+^ cells were co-labeled with GABA (Fig. 2C). The ratio of Glutamate^+^  + c-Fos^+^ to c-Fos^+^ was 81.75% (Fig. 2B), and the ratio of GABA^+^  + c-Fos^+^ to c-Fos^+^ was 11.83% in ACC of CIBP rats (Fig. 2D). These data indicated that glutamatergic neurons were the main type of neurons involved in CIBP.

### Optogenetic or chemogenetic inhibition of ACC glutamatergic neurons promoted PWT in CIBP rats, whereas optogenetic or chemogenetic activation of ACC glutamatergic neurons attenuated PWT in sham rats

**Fig. 3:**
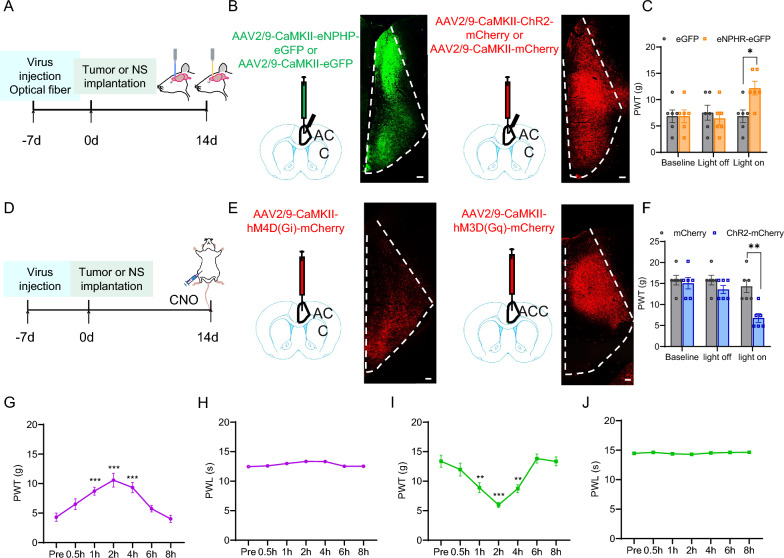
Optogenetic or chemogenetic inhibition of ACC glutamatergic neurons relieved mechanical hyperalgesia in CIBP rats, optogenetic or chemogenetic activation of ACC glutamatergic neurons induced mechanical hyperalgesia in sham rats. **A**, **D** Schematic representation of optogenetic or chemogenetic experiment design. **B** Schematic diagram of the stereotaxic delivery of AAV and fiber into the ACC of CIBP rats and representative images of AAV2/9-CaMKII-eNPHR-eGFP (left) or AAV2/9-CaMKII-ChR2-mCherry (right) expression in the ACC, Scale bar: 100 μm, 10 × magnification. **C** Yellow-light significantly elevated the PWT in CIBP rats transfected with eNPHR-eGFP, but not in rats transfected with eGFP in glutamatergic neurons (n = 6, *p < 0.05, two-way ANOVA followed by Sidak’s multiple comparison test). **E** Schematic diagram of the stereotaxic delivery of AAV into the ACC of CIBP rats and representative images of AAV-CaMKII-hM4D(Gi)-mCherry (left) or AAV-CaMKII-hM3D(Gq)-mCherry (right) expression in the ACC, Scale bar: 100 μm, 10 × magnification. **F** Blue-light significantly lowered the PWT in sham rats transfected with ChR2-mCherry, but not in rats transfected with mCherry in glutamatergic neurons (n = 6, **p < 0.01, two-way ANOVA followed by Sidak’s multiple comparison test). **G**, **I** The effects of PWT after 0.5 h, 1 h, 2 h, 4 h, 6 h and 8 h after CNO injection in rats that received injections of AAV-CaMKII-hM4Di-mCherry or AAV-CaMKII-hM3D(Gq)-mCherry in the ACC (n = 9, **p < 0.01, ***p < 0.001, two-tailed *t*-test). All time points compared to the Pre control group. **H**, **J** The effects of PWL after 0.5 h, 1 h, 2 h, 4 h, 6 h and 8 h after CNO injection in rats that received injections of AAV-CaMKII-hM4Di-mCherry or AAV-CaMKII-hM3D(Gq)-mCherry in the ACC (n = 9, *p* > 0.05, two-tailed *t*-test). All time points compared to the Pre control group

To further verify the role of ACC glutamatergic neurons in CIBP, we used optogenetic and chemogenetic technologies in vivo to selectively and temporarily inhibit glutamatergic neurons. The experimental procedure is depicted in Fig. 3A and D. We injected AAV2/9-CaMKII-eNPHR-eGFP or AAV2/9-CaMKII-eGFP as a control into the ACC of CIBP rats to inhibit glutamatergic neurons (Fig. 3B, Left). The results showed that inhibition of ACC glutamatergic neurons by yellow-light significantly produced an acute and rapid attenuated in mechanical hyperalgesia in CIBP rats (Fig. 3C). Meanwhile, we injected AAV-CaMKII-hM4D(Gi)-mCherry into the ACC of CIBP rats to inhibit glutamatergic neurons (Fig. 3E, Left). We found that 1 h, 2 h and 4 h after CNO injection significantly attenuated mechanical hyperalgesia in CIBP rats (Fig. 3G). However, there was no significant effect in thermal hyperalgesia (Fig. 3H).

Furthermore, we injected AAV2/9-CaMKII-ChR2-mCherry or AAV2/9-CaMKII-mCherry as a control into the ACC of sham rats to activate glutamatergic neurons (Fig. 3B Right). The results showed that blue-light activation of the ACC glutamatergic neurons expressing ChR2-mCherry, but not those expressing mCherry, induced marked mechanical hyperalgesia, as manifested by a drastic decrease in PWT in sham rats (Fig. 3F). At the same time, we injected AAV-CaMKII-hM3D(Gq)-mCherry into the ACC of sham rats to activate glutamatergic neurons (Fig. 3E Right). We found that PWT was decreased from 1 to 4 h after CNO injection (Fig. 3I). However, there was no significant effect in thermal hyperalgesia (Fig. 3J). Consequently, these data indicated that ACC glutamatergic neurons contributed to mechanical hyperalgesia in CIBP rats.

### Calcium activity of ACC glutamatergic neurons was increased in CIBP rats

**Fig. 4:**
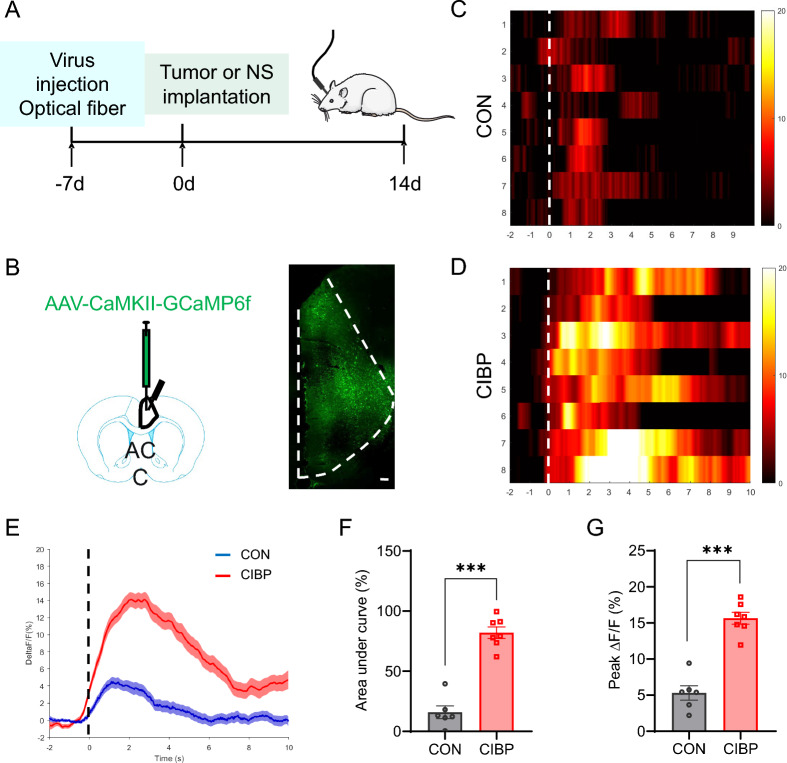
Calcium activity in ACC glutamatergic neurons was activated by CIBP. **A** Schematic representation of in vivo fibro photometry experiment design. **B** Schematic of the stereotaxic delivery of AAV and fiber into the ACC (left) and representative images of AAV-CaMKII-GCaMP6f expression in the ACC (right), Scale bar: 100 μm, 10 × magnification. **C**, **D** Heatmap (left) and average Ca^2+^ signals (ΔF/F) (right) of ACC glutamatergic neurons in CON (upper) and CIBP (lower) rats. **E** Average Ca^2+^ signals (ΔF/F) of ACC glutamatergic neurons in CON (blue) and CIBP (red) rats (CON, n = 6; CIBP, n = 7). **F** Average area under the curve (AUC) of calcium activity of ACC glutamatergic neurons in CON and CIBP rats (CON, n = 6; CIBP, n = 7, ***p < 0.001, two-tailed* t*-test). **G** Averaged peak ΔF/F of calcium activity of ACC glutamatergic neurons in CON and CIBP rats (CON, n = 6; CIBP, n = 7, ***p < 0.001, two-tailed *t*-test)

To investigate dynamic activity of glutamatergic neurons in ACC of CIBP rats, we recorded their Ca^2+^ levels in awake behaving rats using in vivo fibro photometry. Rats were stereotactically injected with AAV-CaMKII-GCaMP6f into ACC. One weeks after virus injection, rats were subjected to CIBP or sham-operation (Fig. 4A, B). We found that a suprathreshold stimulation by von Frey filament stimulation induced a dynamic Ca^2+^ fluctuation in the ACC region of both CIBP and sham rats (Fig. 4C, D). However, the same suprathreshold stimulation triggered a sharp increase in Ca^2^⁺ levels of ACC glutamatergic neurons in CIBP rats compared to the CON group (Fig. 4E–G). These results confirmed that Ca^2+^ activity in ACC glutamatergic neurons were selectively activated by CIBP.

### Surface GluR2 receptors were decreased in the ACC of CIBP rats

**Fig. 5:**
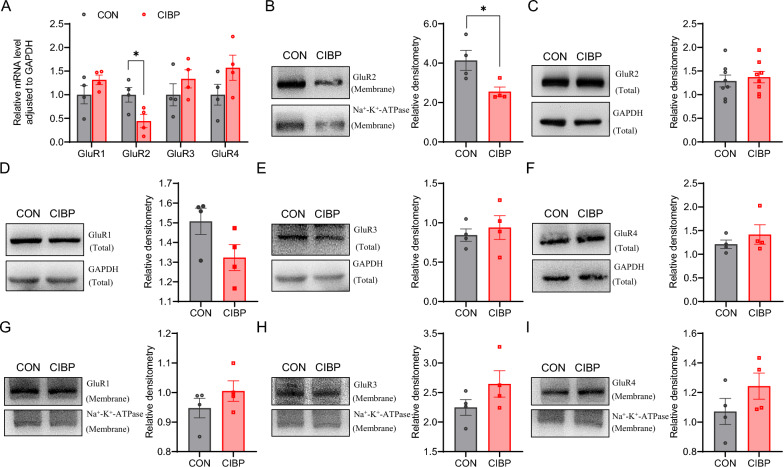
Alteration of surface AMPAR subunits in ACC of CIBP rats. **A** The mRNA level of GluR1 - 4 in left ACC of CIBP rats (n = 4 for each group, *p < 0.05, two-tailed* t*-test). **B** The membrane protein expression of GluR2 in left ACC of CIBP rats (n = 4 for each group, *p < 0.05, two-tailed* t*-test). **C**–**F** The total protein expression of GluR1, GluR2, GluR3 and GluR4 in left ACC of CIBP rats (n = 4 for each group, *p* > 0.05, two-tailed *t*-test). **G**–**I** The membrane protein expression of GluR1, GluR3 and GluR4 in left ACC of CIBP rats (n = 4 for each group, *p* > 0.05, two-tailed *t*-test). The left is representative of Western blots and the right is statistical plots for the relative intensity, GAPDH or Na^+^-K^+^-ATPase is used as an internal control

The levels of GluR1 - 4 mRNA in ACC of CIBP rats were detected by qPCR. Compared with the CON rats, the GluR2 mRNA in the left ACC of CIBP rats was reduced (Fig. 5A). At the level of total protein, there was no significant alteration observed in the expression of GluR1 - 4 receptors in the left ACC between the CIBP and CON groups (Fig. 5C–F). However, at the level of membrane proteins, the expression of GluR2 receptor in the left ACC was reduced in the CIBP group compared to the CON group (Fig. 5B), while there were no significant changes observed in the expression of GluR1, GluR3 and GluR4 receptors (Fig. 5G–I).

### Overexpression of GluR2 alleviated mechanical hypersensitivity in CIBP rats

**Fig. 6:**
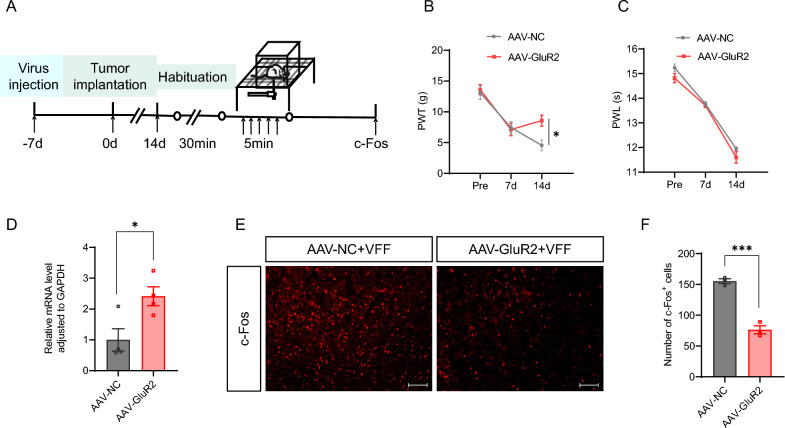
GluR2 overexpression in ACC glutamatergic neurons attenuated mechanical hyperalgesia in CIBP rats. **A** Schematic representation of c-Fos expression evoked by von Frey filament stimulation. **B** Compared with AAV-CaMKII-NC group, PWT in the ipsilateral hind-paw of the AAV-CaMKII-GluR2 group was increased at 14 d after modeling (n = 8 for each group, *p < 0.05, two-way ANOVA followed by Sidak’s multiple comparison test). **C** Compared with AAV-CaMKII-NC group, PWL in the ipsilateral hind-paw of the AAV-CaMKII-GluR2 group was not different at 7 d, 14 d after modeling (n = 8 for each group, *p* > 0.05, two-way ANOVA followed by Sidak’s multiple comparison test). **D** GluR2 mRNA level in ACC of AAV-CaMKII-GluR2 group was upregulated compared with AAV-CaMKII-NC group (n = 4 for each group, *p < 0.05, two-tailed *t*-test). **E** Representative images of c-Fos^+^ neurons in left ACC after von Frey filament stimulation. Scale bar:100 μm, 10 × magnification. **F** Number of c-Fos^+^ cells in ACC after von Frey filament stimulation (n = 3 for each group, ***p < 0.001, two-tailed *t*-test)

To validate the impact of GluR2 on CIBP, we injected a virus that overexpresses GluR2 or a control virus into the left ACC of the rats. Compared to the AAV-CaMKII-NC group, the AAV-CaMKII-GluR2 group exhibited a significant upregulation in the mRNA level of GluR2, thereby indicating the efficacy of GluR2-overexpressing virus (Fig. 6D). The experimental results demonstrated that there was no significant change in the PWT of rats in the AAV-CaMKII-GluR2 group on 7 d, compares to the AAV-CaMKII-NC group; however, a significant increase was observed on 14 d (Fig. 6B). Furthermore, the PWL remained unchanged in the AAV-CaMKII-GluR2 group at both 7 d and 14 d (Fig. 6C). Meanwhile, in comparison to the AAV-CaMKII-NC group, the AAV-CaMKII-GluR2 group exhibited a significant reduction in the number of c-Fos^+^ cells in the left ACC (Fig. 6A, E, F). These results indicate that decreased expression of the GluR2 receptors contributed to mechanical hyperalgesia, and GluR2 overexpression alleviated mechanical hypersensitivity in CIBP rats.

### GluR2 receptors were mainly expressed in ACC glutamatergic neurons and negatively regulated mechanical hypersensitivity

**Fig. 7:**
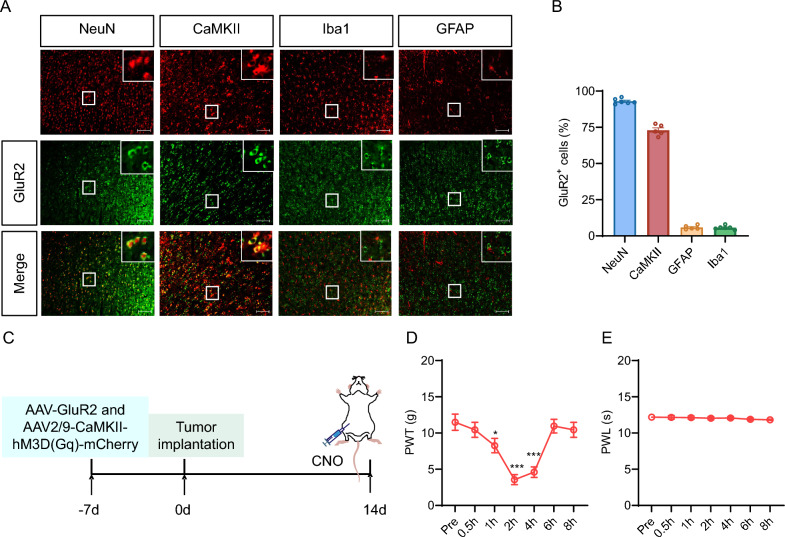
GluR2 receptors were mainly expressed in ACC glutamatergic neurons and negatively regulated mechanical hyperalgesia. **A** Representative images of co-localization of GluR2 (green) with NeuN (red), CaMKII (red), GFAP (red), or Iba1 (red) in ACC of normal rats, Scale bar:100 μm, 10 × magnification. The protein marker NeuN was expressed in the nucleus, whereas CaMKII, Iba1, and GFAP were expressed in the cytoplasm. **B** Quantified analysis showed GluR2 was mainly present in NeuN-positive neurons and CaMKII-positive neurons with a very small amount in GFAP-positive astrocytes and Iba1-positive microglial cells in ACC. **C** Schematic representation of experiment design. **D** The effects of PWT after 0.5 h, 1 h, 2 h, 4 h, 6 h and 8 h after CNO injection in AAV-CaMKII-GluR2 + hM3D group that received injections of AAV-CaMKII-hM3D(Gq)-mCherry and AAV-CaMKII-GluR2 in the ACC (n = 8, *p < 0.05, *** *p* < 0.001, two-tailed* t*-test). **E** The effects of PWL after 0.5 h, 1 h, 2 h, 4 h, 6 h and 8 h after CNO injection in AAV-CaMKII-GluR2 + hM3D group that received injections of AAV-CaMKII-hM3D(Gq)-mCherry and AAV-CaMKII-GluR2 in the ACC (n = 8, *p* > 0.05, two-tailed *t*-test)

We investigated the distribution of GluR2 receptors in the ACC of normal rats by examining their co-localization with various cellular markers. The results showed that the GluR2 receptors were mainly co-expressed with NeuN (Neuronal nuclei) and glutamatergic neuron marker CaM-dependent protein kinase II (CaMKII), but they were not associated with Glial fibrillary acidic protein (GFAP) in astrocyte, and Ionized calcium binding adapter molecule 1 (Iba1) in microglia (Fig. 7A, B). These results suggested that GluR2 receptors were mainly expressed in ACC glutamatergic neurons. In order to further verify the effects of GluR2 receptors in ACC glutamatergic neurons on PWT and PWL in CIBP rats, we injected GluR2 overexpression virus and AAV-CaMKII-hM3D(Gq)-mCherry virus (AAV-CaMKII-GluR2 + hM3D group) into the left ACC of rats (Fig. 7C). The results showed that the PWT of CIBP rats in AAV-CaMKII-GluR2 + hM3D group was reduced at 1 h, 2 h and 4 h after the administration of CNO-activated glutamatergic neurons (Fig. 7D), but the PWL had no significant change (Fig. 7E).

## Discussion

Cancer-induced bone pain (CIBP) is a severe clinical condition that occurs when cancer cells spread to the spinal cord and bone tissue (Lin, et al. 2022). The pathophysiology of CIBP is complex, involving inflammatory factors, neuropathic components, and cancer-specific mediators (Cao et al. 2010). Current pharmacological treatments for CIBP encompass nonsteroidal anti-inflammatory drugs (NSAIDs), potent opioid analgesics, bisphosphonates, and monoclonal antibodies targeting osteoclast activity (Jing et al. 2023). While opioids are recommended as the first-line therapy by the World Health Organization (WHO), their use can be limited by adverse effects like gastrointestinal symptoms, fatigue, decreased consciousness, and respiratory depression (Sindhi and Erdek 2019). Even with adherence to WHO guidelines, around 10–20% of CIBP patients still do not experience relief (Christo and Mazloomdoost 2008). Therefore, understanding the pathophysiological mechanisms of CIBP and identifying new treatment targets are crucial. This study established a CIBP model in rats by injecting Walker 256 cells into the tibial bone marrow cavity, closely mimicking bone cancer pain from human breast cancer bone metastasis (Segelcke, et al. 2023; Yang et al. 2021; Diaz-delCastillo, et al. 2020; Liu et al. 2021; Ge, et al. 2022).

The results of PWT and PWL in the ipsilateral hind-paw of the CIBP group showed a gradual decrease as CIBP progressed. while no significant changes were observed in the contralateral hind-paw, the results indicate that the CIBP model has been successfully established. The ACC has been implicated in sensory processing, including pain perception (Tu 2023), as well as motor and cognitive functions (Seamans and Floresco 2022). Early study revealed that cingulotomy provided pain relief to 60% of patients with advanced cancer or non-malignant tumors (Ballantine et al. 1967). However, the specific role of ACC in various pain states, particularly CIBP, remains underexplored. By utilizing the immunofluorescence technique, a significant augmentation in the quantity of c-Fos^+^ neurons within the ACC were observed in rats experiencing CIBP compared to those in the control group. The c-Fos^+^ neurons were found to be co-localized with Glutamate-positive neurons This suggests a pivotal role of ACC glutamatergic neurons in CIBP regulation. Further validation was done through optogenetic and chemogenetic techniques, showing that inhibition of glutamatergic neurons in the ACC led to pain relief in CIBP rats, while activation resulted in increased pain perception in control rats. Additionally, in vivo fibro photometry revealed a significant increase in calcium activity of ACC glutamatergic neurons in CIBP rats. These findings collectively demonstrate ACC glutamatergic neurons contribute CIBP and their significant role in pain modulation.

Glutamate is the primary excitatory neurotransmitter in the mammalian cerebral cortex (Petroff 2002). Glutamate receptors are classified into two main categories: ionotropic glutamate receptors (iGluRs) and metabotropic glutamate receptors (mGluRs) (Henley and Wilkinson 2016). AMPA receptors, a type of iGluR, have been shown through pharmacological, electrophysiological, and behavioral studies to play a vital role in regulating neuronal excitability and synaptic transmission in the central nervous system (Bleakman et al. 2006). Specifically, AMPA receptors are crucial in transmitting pain signals. These receptors consist of subunits GluR1, GluR2, GluR3, and GluR4 (Zanetti, et al. 2021). The GluR2 subunit is particularly important, influencing the assembly, transport, and various forms of long-term potentiation (LTP) (Isaac et al. 2007). In the brain, most GluR2 exists in a Q/R-edited form that blocks Ca^2+^ permeation (Verdoorn, et al. 1991). Thus, AMPA receptors containing edited GluR2 subunits exhibit impermeability to Ca^2+^, whereas those lacking GluR2 or possessing unedited GluR2 are permeable to Ca^2+^ (Hestrin 1993). Studies have linked GluR2 receptors at synapses to the development and progression of neuropathic pain, aversion, and depression following spinal cord nerve ligation (SNL) in rats (Jiang et al. 2021). The study examined the potential involvement of AMPA receptors in the development and maintenance of CIBP. RT-qPCR technology was used to detect the mRNA level of AMPA receptors in the left ACC of rats with CIBP. The results showed a significant decrease in GluR2 receptor mRNA levels, which was further confirmed by Western blotting demonstrating a reduction in GluR2 receptor membrane protein levels. These findings suggest that GluR2 receptors negatively regulate the occurrence and progression of CIBP. Immunofluorescence staining was then utilized to show the co-expression of GluR2 receptors with glutamatergic neurons, indicating that the downregulation of GluR2 receptors in ACC glutamatergic neurons'postsynaptic membranes may enhance Ca^2+^ permeability and increase synaptic excitability, ultimately leading to heightened pain sensitivity in rats with CIBP.

To further investigate the impact of GluR2 receptor on CIBP, we conducted experiments where AAV virus overexpressing GluR2 receptor was administered into the left ACC of CIBP rats. The results showed a significant decrease in c-Fos expression within their ACC. Behavioral tests revealed a notable increase in PWT following the microinjection of AAV-CaMKII-GluR2 into the left ACC, although no significant change was observed in PWL. Subsequent chemogenetic techniques indicated that activating glutamatergic neurons in the left ACC of CIBP rats could reverse the heightened PWT induced by AAV-CaMKII-GluR2 overexpression. These findings suggested that GluR2 overexpression in ACC glutamatergic neurons alleviates cancer-induced bone pain.

c-Fos is a widely used marker for neuronal activation, as its expression is rapidly induced in response to various stimuli, making it a valuable tool for mapping functional activity in the brain. c-Fos is a well-established immediate-early gene that serves as a marker for neuronal activation. The study aims to investigate changes in neural activity in response to pain stimulation, c-Fos expression provides insights into which brain regions are involved. c-Fos expression peaks within 1 h after stimulation and returns to baseline levels within a few hours. When neurons are activated by pain stimuli, intracellular signaling pathways are triggered. Key pathways involved in c-Fos induction include Calcium influx (through voltage-gated calcium channels or NMDA receptors), second messenger systems (cAMP, MAPK/ERK, or PKC), transcription factor activation (CREB) is phosphorylated and binds to the c-Fos promoter to initiate transcription. The c-Fos protein forms a heterodimer with Jun family proteins to create the AP- 1 transcription factor complex. The AP- 1 complex binds to DNA at specific promoter regions to regulate the expression of downstream target genes. These target genes may be involved in synaptic plasticity, neuronal growth and differentiation.

This study also has some limitations that need to be considered. Firstly, we injected viruses in rat ACC to overexpress GluR2 to relieve bone cancer pain, a method that can be used for animal mechanism studies but is difficult to use in clinical treatment. Secondly, Optogenetic or chemogenetic inhibition of ACC glutamatergic neurons attenuated mechanical hyperalgesia in CIBP rats. This method is traumatic to the brain and can be used in animal studies, but is restricted in clinical treatment. Thirdly, the sample size of this study is relatively small and more samples, models and methods are needed to confirm the role of GluR2 overexpression or agonist in the treatment of bone cancer pain.

## Conclusions

**Fig. 8:**
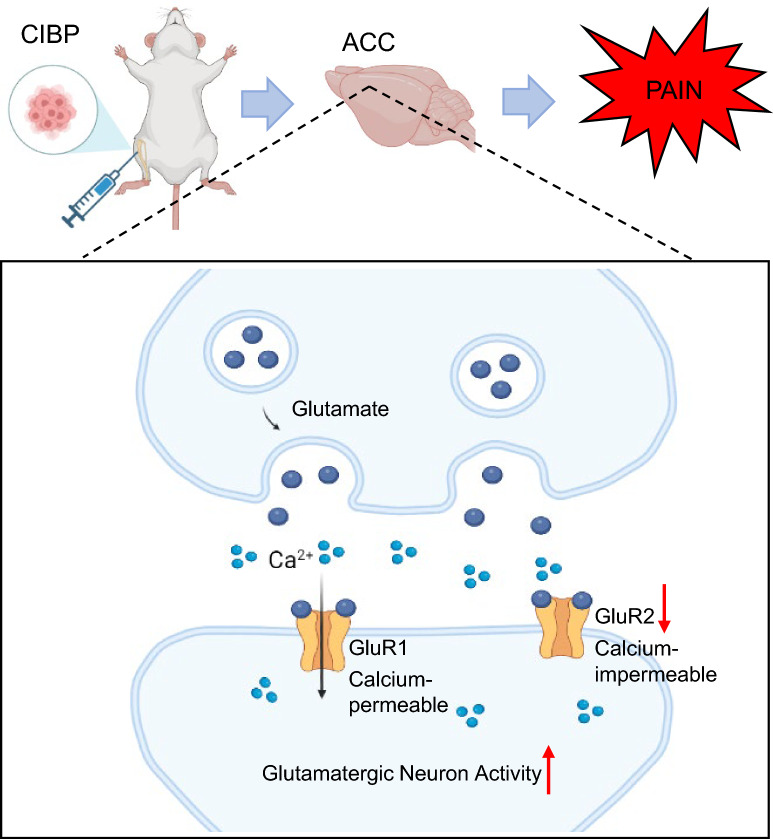
Reducing surface GluR2 in ACC glutamatergic neurons causes cancer-induced bone pain in rats

This study demonstrates that reducing surface GluR2 receptors in ACC glutamatergic neurons contribute to pain hypersensitivity in rats with CIBP, while overexpressing GluR2 receptors in ACC glutamatergic neurons could alleviate pain hypersensitivity in rats with CIBP (Fig. 8).

## Data Availability

All data generated or analyzed during this study are included in this published article. The datasets used or analyzed during the current study are available from the corresponding author upon reasonable request.
